# Becoming More CAPABLE: Preliminary Results of Implementing a Person-Centered Intervention for Aging in Place

**DOI:** 10.1093/geroni/igaf122.991

**Published:** 2025-12-31

**Authors:** Pamela Toto, Ava Giatras, Kate Perepezko, Morgan Blanchflower, Beth Fields

**Affiliations:** University of Pittsburgh, Pittsburgh, Pennsylvania, United States; University of Pittsburgh, Pittsburgh, Pennsylvania, United States; Miami University, Oxford, Ohio, United States; University of Pittsburgh, Pittsburgh, Pennsylvania, United States; University of Wisconsin–Madison, Madison, Wisconsin, United States

## Abstract

Community Aging in Place, Advancing Better Living for Elders (CAPABLE) is an evidence-based intervention that combines occupational therapy, nursing, and low-cost home modifications to target “what matters” to community older adults. CAPABLE has been demonstrated to promote aging in place by delaying disability, preventing hospitalizations, and avoiding institutionalization, yet limited information exists on its real-world application through existing home and community-based services (HCBS). This study descibed preliminary results of a pilot providing CAPABLE through an area agency on aging (AAA). An effectiveness-implementation hybrid trial type 1 study was completed implementing CAPABLE with 90 older adults. Participants were recruited through 3 HCBS agencies affiliated with an AAA and received up to 10 visits and $1300 in home modifications over a 5-month period to address self-identified functional goals. Descriptive statistics were conducted to assess the program’s feasibility, cost, and acceptability. 69% (n = 62) participants completed CAPABLE. Primary reasons for non-completion included health decline, death, or move to a skilled nursing facility. On average, participants identified and set 4.5 goals (range 1 -6). Most common goal areas included bathing, functional mobility, and pain. Home modification costs averaged $943 per participant with most common modifications including grab bars (33%), railings (16%) and lighting (15%). Program satisfaction scores indicated high satisfaction with # of visits, length of program, home modifications and goal attainment. Preliminary outcomes suggest that delivering CAPABLE through an AAA and HCBS is a feasible and acceptable approach to supporting older adults in achieving self-identified goals for aging in place through low-cost home modifications.

